# Supplementary material for: Sexual conflict drives micro- and macroevolution of sexual dimorphism in immunity
Source: BMC Biol. 2021 Jun 2;19:114. doi: 10.1186/s12915-021-01049-6 (PMC8170964; doi:10.1186/s12915-021-01049-6)
Supplement: Supplementary file 2 — Additional file 2: Supplement 1. Optimization of PO-activity assays. Figure S1. Test kinetics of the phenoloxidase assays. Table S1. PO-activity after preincubation of frozen homogenate with different activators. Supplement 2. Mating status and phenotypic plasticity of PO-activity in females. Table S2. ANOVA on the effects of mating treatment and egg laying on PO-activity. Supplement 3. Experimental Evolution of PO-activity. Table S3a. Effects of mating treatment and mating regime on adult weight. Table S3b. Analysis of differences in offspring production between evolution regimes. Table S3c. Effects of mating treatment and mating regime on male PO-activity. Supplement 4. Responses to bacterial infection in the experimental evolution lines. Table S4a. Effect of infection with B. thuringiensis on survival of virgin and mated females from polygamous and monogamous regimes. Table S4b.1 Effect of infection with B. thuringiensis on survival of virgin and mated females from polygamous and monogamous regimes, using a binomial response. Table S4c. Effect of bacterial infection on survival of virgin males from polygamous and monogamous regimes. Table S4d. Effect of infection with P. entomophila on survival of mated females from polygamous and monogamous mating regimes. Table S4e. Effect of infection with P. entomophila on survival of mated females from polygamous and monogamous mating regimes using a binomial response. Table S4f. Bacterial load of P. enthomophila in mated females from polygamous and monogamous regimes. Supplement 5. Macroevolutionary change and sexually antagonistic coevolution between male genital morphology and female PO-activity. Figure S5a. Macroevolutionary change in sexual dimorphism in PO-activity across seed beetle linages. Figure S5b. Photos of genitalia rated as least and most harmful. Table S5a PGLS of male genital morphology and female PO-activity. Table S5b. PGLS of male genital morphology and male PO-activity. [file 12915_2021_1049_MOESM2_ESM.docx]

**Additional File 2**

**Sexual conflict drives micro- and macroevolution of sexual dimorphism in immunity**

*Basabi Bagchi^1*^, Quentin Corbel^2,3^*, Imroze Khan^1*^, Ellen Payne^2^*, Devshuvam Banerji^1^, Johanna Liljestrand-Rönn^2^, Ivain Martinossi-Allibert^2,5^, Julian Baur^2^, Elina Immonen^,4^, Ahmed Sayadi^2,6^, Göran Arnqvist^2^, Irene Söderhäll^7^ & David Berger^2^†.*

† Corresponding author: [david.berger@ebc.uu.se](mailto:david.berger@ebc.uu.se)

*Equal contribution and presented in alphabetical order

**Supplement 1:** Optimization of PO-activity assays

**Supplement 2:** Mating status and phenotypic plasticity of PO activity in females

**Supplement 3:** Microevolution of PO activity.

**Supplement 4:** Responses to bacterial infection in the experimental evolution lines

**Supplement 5:** Macroevolutionary change and coevolution between male genital morphology and female PO activity.

**Supplement 1: Optimization of PO-activity assays**

**Figure S1:** In order to test kinetics of the phenoloxidase assay, we prepared samples by mixing homogenates from three beetles, incubating as described in the Material and Methods, and followed absorbance at 420 nm each minute for 25 min to check linearity of the reaction. This experiment was repeated with three different homogenates, and the result is shown in the figure below. In addition, one pooled sample was preincubated with phenylthiourea (PTU) at 10 mM final concentration.

To test if all proPO was converted to PO in the frozen homogenates used in our experiments, the activity of pooled samples from three females were tested for activity after preincubation with curdlan (a β-1,3-glucan) at 1 mg/mL, or trypsin (0,1 mg/mL) or chymotrypsin (0,1 mg/mL). As shown in the table below, no additional activation could be observed after freezing the homogenates. We note that when assaying PO activity in fresh (unfrozen) samples, it is important to always test the presence of inactive proPO, since in a few fresh homogenates some minor increased PO-activity after preincubation with trypsin or chymotrypsin was found, indicating presence of intact proPO.

**Table S1:** PO-activity after preincubation of frozen homogenate with different activators.

|  | Sample A | Sample B | Sample C |
| --- | --- | --- | --- |
| Treatment | ΔA420/min | ΔA420/min | ΔA420/min |
| No activation | 0,0382 | 0,0429 | 0,0223 |
| Trypsin | 0,0368 | 0,0428 | 0,0217 |
| Chymotrypsin | 0,0375 | 0,0435 | 0,0197 |
| Curdlan | 0,0377 | 0,0433 | 0,0203 |

**Supplement 2: Mating status and phenotypic plasticity of PO-activity in females**

**Table S2:** ANOVA on the effects of mating treatment and egg laying on PO-activity.

lm(PO ~ weight+treatment*eggs,na.action=na.omit, data=mate)->mod2

Anova Table (Type II tests)

Response: PO

Sum Sq Df F value Pr(>F)

weight 0.0005784 1 1.9198 0.1722

treatment 0.0168899 3 18.6867 3.229e-08 ***

eggs 0.0000252 1 0.0836 0.7737

treatment:eggs 0.0009753 3 1.0790 0.3667

Residuals 0.0147628 49

**Supplement 3: Experimental Evolution of PO-activity.**

**Table S3a:** Model specification and summary for analyses of effects of mating treatment and mating regime on adult weight.

prior1 = list(R = list(V = 0.001, nu = 0.002), G = list(G1 = list(V = diag(0.001,4), nu = 5), G2 = list(V = 0.001, nu = 0.002)))

modAW <- MCMCglmm(log(aw) ~ treat*sex*Regime + date2,

random = ~us(treat:sex):line + dish, data = immunity,

family = "gaussian",prior = prior1, nitt=110000,slice=TRUE, burnin=10000, thin=100, verbose = FALSE)

DIC: -660.5602

G-structure: ~us(treat:sex):line

post.mean l-95% CI u-95% CI eff.samp

M:sexf:M:sexf.line 2.716e-03 0.0002763 0.007739 1000

atV:sexf:M:sexf.line -8.429e-05 -0.0029372 0.003751 1000

M:sexm:M:sexf.line -2.319e-04 -0.0040262 0.002912 1000

atV:sexm:M:sexf.line -8.202e-05 -0.0033890 0.003592 1000

M:sexf:atV:sexf.line -8.429e-05 -0.0029372 0.003751 1000

atV:sexf:atV:sexf.line 2.486e-03 0.0003563 0.006842 1000

M:sexm:atV:sexf.line 1.590e-06 -0.0035273 0.003349 1000

atV:sexm:atV:sexf.line 3.451e-06 -0.0031724 0.003331 1000

M:sexf:M:sexm.line -2.319e-04 -0.0040262 0.002912 1000

atV:sexf:M:sexm.line 1.590e-06 -0.0035273 0.003349 1000

M:sexm:M:sexm.line 2.963e-03 0.0003904 0.007973 1000

atV:sexm:M:sexm.line 4.087e-04 -0.0028881 0.004381 1000

M:sexf:atV:sexm.line -8.202e-05 -0.0033890 0.003592 1000

atV:sexf:atV:sexm.line 3.451e-06 -0.0031724 0.003331 1000

M:sexm:atV:sexm.line 4.087e-04 -0.0028881 0.004381 1000

atV:sexm:atV:sexm.line 2.667e-03 0.0003275 0.007091 1000

~dish

post.mean l-95% CI u-95% CI eff.samp

dish 0.001222 3.774e-07 0.002554 571.2

R-structure: ~units

post.mean l-95% CI u-95% CI eff.samp

units 0.0222 0.01979 0.0249 1000

Location effects: log(aw) ~ treat * sex * evol + date2

post.mean l-95% CI u-95% CI eff.samp pMCMC

Intercept(male-lim) -5.600057 -5.687583 -5.516414 1146.7 <0.001 ***

treatVirgin 0.235430 0.130255 0.354929 1254.3 0.004 **

sex.m -0.425792 -0.557175 -0.316267 739.9 <0.001 ***

evol.mono 0.086984 -0.033215 0.195206 1102.3 0.136

evol.poly -0.004587 -0.115949 0.107136 1000.0 0.936

date2 batch2 -0.009607 -0.055843 0.030553 1000.0 0.636

treatV:sexm -0.021025 -0.158023 0.128074 1000.0 0.802

treatV:evolmono 0.030475 -0.131892 0.208241 1070.6 0.698

treatV:evolpoly 0.061962 -0.095706 0.205128 1183.9 0.408

sexm:evolmono -0.029061 -0.186639 0.145714 877.7 0.700

sexm:evolpoly 0.025689 -0.132726 0.192904 1000.0 0.730

treatV:sexm:evolmono -0.011103 -0.251075 0.207852 797.6 0.898

treatV:sexm:evolpoly -0.003060 -0.208250 0.204767 1000.0 0.930

**Table S3b:** Model specification and summary for analyses of effects of mating treatment and mating regime on male PO-activity.

prior1a = list(R = list(V = 1, nu = 10^-6),

G = list(G1 = list(V = 1, nu = 10^-6),G2 = list(V = 1, nu = 10^-6),

G3 = list(V = 1, nu = 10^-6),G4 = list(V = 1, nu = 10^-6)))

modPO1 <- MCMCglmm(POcorr*100 ~ treat*Regime*scale(aw) + date2 + frozen.alive,

random = ~line + treat:line + aw:line + treat:aw:line, data = imM,

family = "gaussian",prior = prior1a, nitt=3100000,slice=TRUE, burnin=100000, thin=3000, verbose = FALSE)

DIC: 799.2401

G-structure: ~line

post.mean l-95% CI u-95% CI eff.samp

line 0.05037 9.353e-08 0.09085 1000

~treat:line

post.mean l-95% CI u-95% CI eff.samp

treat:line 0.01622 1.069e-07 0.07501 1000

~aw:line

post.mean l-95% CI u-95% CI eff.samp

aw:line 0.0164 2.07e-07 0.1064 1000

~treat:aw:line

post.mean l-95% CI u-95% CI eff.samp

treat:aw:line 0.04399 8.409e-08 0.2114 600.3

R-structure: ~units

post.mean l-95% CI u-95% CI eff.samp

units 0.4959 0.324 0.6379 1000

Location effects: POcorr * 100 ~ treat * Regime * scale(aw) + date2 + frozen.alive

post.mean l-95% CI u-95% CI eff.samp pMCMC

Intercept (monogamy) -0.92479 -1.34161 -0.49606 1000.0 0.002 **

treatmated 0.29644 -0.19470 0.69702 1000.0 0.200

Regime.poly 0.39662 -0.12329 0.96676 1000.0 0.118

Regime.male -0.03171 -0.56118 0.55625 1000.0 0.872

scale(aw) 0.04250 -0.23234 0.33188 1000.0 0.768

date2batch2 -0.04258 -0.39569 0.28392 1000.0 0.740

frozen.aliveN2 0.33676 -0.13060 0.79397 1211.1 0.144

frozen.aliveY 0.06616 -0.18781 0.33586 1000.0 0.628

treatmated:Regimepoly -0.40597 -0.98814 0.26107 1000.0 0.182

treatmated:Regimemale 0.04196 -0.60943 0.65505 1000.0 0.892

treatmated:scale(aw) -0.24605 -0.64610 0.16304 1000.0 0.272

Regimepoly:scale(aw) -0.17127 -0.54120 0.22086 1000.0 0.368

Regimemale:scale(aw) -0.17745 -0.53124 0.24447 1000.0 0.362

treatmated:Regimepoly:scale(aw) 0.19683 -0.38369 0.72463 832.3 0.526

treatmated:Regimemale:scale(aw) 0.31375 -0.24755 0.82759 701.9 0.268

**Table S3c:** Analysis of differences in offspring production between evolution regimes in the 46h mating treatment using a mixed effect model based using REML estimation.

lmer(offspring/5 ~ evolution + date2 + (1|line), data=fec[1:37,]) -> mod

REML criterion at convergence: 214.8

Random effects:

Groups Name Variance Std.Dev.

line (Intercept) 0.00 0.000

Residual 33.81 5.814

Number of obs: 37, groups: line, 6

Fixed effects:

Estimate Std. Error t value

Intercept (male) 52.210 2.890 18.065

Evolution.mono -3.813 2.383 -1.600

Evolution.poly 2.677 2.281 1.174

date2batch2 3.338 1.919 1.739

Analysis of Deviance Table (Type II Wald chisquare tests)

Response: offspring/5

Chisq Df Pr(>Chisq)

evolution 7.4380 2 0.02426 *

date2 3.0242 1 0.08203 .

**Supplement 4: Responses to bacterial infection in the experimental evolution lines**

**Table S4a:** Cox-proportional hazards regression estimating the effect of infection with *B. thuringiensis* on survival of virgin and mated females from polygamous and monogamous regimes, following 50 generations of experimental evolution. Censused beyond 10 days post-infection.

cox_10 <- coxme(Surv(time = data2$hours, event = data2$censor) ~ regime*treatment*mating + block + (1|line), data = data2)

Cox mixed-effects model fit by maximum likelihood

Data: data2

events, n = 835, 1060

Iterations= 5 27

NULL Integrated Fitted

Log-likelihood -5330.719 -5248.14 -5248.031

Chisq df p AIC BIC

Integrated loglik 165.16 14.00 0 137.16 70.97

Penalized loglik 165.38 13.04 0 139.29 77.63

Fixed coefficients

coef exp(coef) se(coef) z p

regimeMO 0.148866769 1.1605184 0.21984854 0.68 5.0e-01

treatment1 od 0.879119736 2.4087784 0.19788298 4.44 8.9e-06

treatment2 od 1.182189666 3.2615080 0.19656507 6.01 1.8e-09

mating 0.635735994 1.8884115 0.19699346 3.23 1.3e-03

block2 0.002521189 1.0025244 0.09242857 0.03 9.8e-01

block3 0.027353935 1.0277315 0.08110524 0.34 7.4e-01

regimeMO:treatment1 od -0.636142136 0.5293306 0.28486042 -2.23 2.6e-02

regimeMO:treatment2 od -0.666092543 0.5137120 0.28018858 -2.38 1.7e-02

regimeMO:mating 0.092003510 1.0963687 0.27608750 0.33 7.4e-01

treatment1 od:mating -0.152276477 0.8587508 0.25400174 -0.60 5.5e-01

treatment2 od:mating -0.246807326 0.7812912 0.25190300 -0.98 3.3e-01

regimeMO:treatment1 od:mating 0.130854710 1.1398022 0.36428569 0.36 7.2e-01

regimeMO:treatment2 od:mating 0.110071650 1.1163581 0.35843071 0.31 7.6e-01

Random effects

Group Variable Std Dev Variance

line Intercept 0.0127639889 0.0001629194

Analysis of Deviance Table (Type II tests)

Df Chisq Pr(>Chisq)

regime 1 9.8152 0.001731 **

treatment 2 70.8506 4.121e-16 ***

mating 1 63.6131 1.514e-15 ***

block 2 0.1350 0.934710

regime:treatment 2 13.7176 0.001050 **

regime:mating 1 1.6283 0.201945

treatment:mating 2 1.1752 0.555654

regime:treatment:mating 2 0.1435 0.930786

**Table S4b:** Bayesian mixed effects model estimating the effect of infection with *B. thuringiensis* on survival of virgin and mated females from polygamous and monogamous mating regimes, following 50 generations of experimental evolution. Binomial response estimated after 5 days of census, at which point 51.8% (549/1060) of all females, including controls, were still alive. Interactions with P>0.2 were removed from the model presented below.

#model specification

prior_line = list(R = list(V = 1, fix = 1),

G = list(G1 = list(V = 1, nu =10^-6), G2 = list(V = 1, nu =10^-6)))

MCMC_120 <- MCMCglmm(censor120 ~ mating + regime*treatment,

+ random = ~ line + treatment:line,

+ rcov = ~ units, data = data2,

+ family = "categorical", prior = prior_line, nitt=550000,

+ slice=TRUE, burnin=50000, thin=500)

#model output

Iterations = 50001:549501

Thinning interval = 500

Sample size = 1000

DIC: 1326.432

G-structure: ~line

post.mean l-95% CI u-95% CI eff.samp

line 0.01392 9.763e-08 0.06755 1000

~treatment:line

post.mean l-95% CI u-95% CI eff.samp

treatment:line 0.0054 1.43e-07 0.03166 1000

R-structure: ~units

post.mean l-95% CI u-95% CI eff.samp

units 1 1 1 0

Location effects: censor120 ~ mating + regime * treatment

post.mean l-95% CI u-95% CI eff.samp pMCMC

(Intercept) 1.9563 1.4238 2.5020 1000.0 <0.001 ***

regimeMO -0.2939 -0.9984 0.4853 1000.0 0.452

mating -0.9125 -1.3224 -0.3956 1000.0 <0.001 ***

treatment1 od -2.0138 -2.5720 -1.5103 843.6 <0.001 ***

treatment2 od -2.4411 -3.0202 -1.8552 1000.0 <0.001 ***

regimeMO:treatment1 od 0.9421 0.1318 1.7124 1000.0 0.018 *

regimeMO:treatment2 od 1.2943 0.5221 2.0855 1000.0 <0.001 ***

**Table S4c:** Cox-proportional hazards regression estimating the effect of bacterial infection on survival of virgin males from polygamous and monogamous regimes following 50 generations of experimental evolution. Censused over 5 days following infection, when 35% (176/270) of all males were still alive. There were significant effects of the bacterial infection (P = 0.021) and monogamous males died faster than polygamous males (P = 0.010), but there was no difference in the effect of infection between regimes (P = 0.63).

Cox mixed-effects model fit by maximum likelihood

Data: data

events, n = 176, 270

Iterations= 9 48

NULL Integrated Fitted

Log-likelihood -908.3705 -896.3209 -894.5783

Chisq df p AIC BIC

Integrated loglik 24.10 8.00 0.00220560 8.10 -17.26

Penalized loglik 27.58 7.62 0.00042412 12.34 -11.83

Model: Surv(time = data$hours, event = data$censor) ~ regime * treatment + block + (1 | line)

Fixed coefficients

coef exp(coef) se(coef) z p

regimeMO 0.6977231 2.0091728 0.3042473 2.29 0.022

treatment1OD 0.6146787 1.8490625 0.2953745 2.08 0.037

treatment2.5OD 0.6576819 1.9303126 0.2982778 2.20 0.027

block2 0.2042124 1.2265587 0.2282020 0.89 0.370

block3 0.3967714 1.4870159 0.2282114 1.74 0.082

regimeMO:treatment1OD -0.3668521 0.6929121 0.3834369 -0.96 0.340

regimeMO:treatment2.5OD -0.2485621 0.7799214 0.3848408 -0.65 0.520

Analysis of Deviance Table (Type II tests)

Response: Surv(time = data$hours, event = data$censor)

Df Chisq Pr(>Chisq)

regime 1 6.6288 0.01003 *

treatment 2 7.7733 0.02051 *

block 2 3.0237 0.22051

regime:treatment 2 0.9354 0.62644

**Table S4d:** Cox-proportional hazards regression estimating the effect of infection with *P. entomophila* on survival of mated females from polygamous and monogamous mating regimes following 55 generations of experimental evolution. Censused beyond 5 days post-infection.

Cox mixed-effects model fit by maximum likelihood

Data: data

events, n = 209, 288

Iterations= 14 87

NULL Integrated Fitted

Log-likelihood -1077.392 -1041.207 -1039.683

Chisq df p AIC BIC

Integrated loglik 72.37 7.00 4.8972e-13 58.37 34.97

Penalized loglik 75.42 6.43 5.6510e-14 62.55 41.04

Model: Surv(time = data$hours, event = data$censor) ~ regime * treatment + block + (1 | line)

Fixed coefficients

coef exp(coef) se(coef) z p

regimePolygamy -0.07289093 0.9297022 0.2928655 -0.25 0.8000

treatment0.5OD 0.42975950 1.5368879 0.2530109 1.70 0.0890

treatment1OD 0.47976262 1.6156908 0.2514013 1.91 0.0560

block2 0.26041180 1.2974643 0.1873123 1.39 0.1600

regimePolygamy:treatment0.5OD 0.41907793 1.5205588 0.3562258 1.18 0.2400

regimePolygamy:treatment1OD 1.36338175 3.9093916 0.3511195 3.88 0.0001

Random effects

Group Variable Std Dev Variance

line Intercept 0.12379893 0.01532618

Analysis of Deviance Table (Type III tests)

Response: Surv(time = data$hours, event = data$censor)

Df Chisq Pr(>Chisq)

regime 1 0.0619 0.8034469

treatment 2 4.2609 0.1187816

block 1 1.9328 0.1644516

regime:treatment 2 16.5703 0.0002522 ***

**Table S4e:** Bayesian mixed effects model estimating the effect of infection with *P. enthomophila* on survival of mated females from polygamous and monogamous mating regimes following 55 generations of experimental evolution. Binomial response estimated after 72h of census, at which point ca. 50% of all females, including controls, were still alive.

prior_line = list(R = list(V = 1, fix = 1),

+ G = list(G1 = list(V = 1, nu =10^-6), G2 = list(V = 1, nu =10^-6)))

MCMC_3 <- MCMCglmm(censor72 ~ regime*treatment + block,

+ random = ~ line + treatment:line,

+ rcov = ~ units, data = data.frame(data),

+ family = *"categorical"*, prior = prior_line,

+ nitt=1050000,

+ slice=TRUE, burnin=50000, thin=500, verbose = FALSE,

+ pr=F)

Iterations = 50001:1049501

Thinning interval = 500

Sample size = 2000

DIC: 328.3504

G-structure: ~line

post.mean l-95% CI u-95% CI eff.samp

line 1139 7.705e-08 10.32 2000

~treatment:line

post.mean l-95% CI u-95% CI eff.samp

treatment:line 0.2049 1.306e-07 0.977 1818

R-structure: ~units

post.mean l-95% CI u-95% CI eff.samp

units 1 1 1 0

Location effects: censor72 ~ regime * treatment + block

post.mean l-95% CI u-95% CI eff.samp pMCMC

(Intercept) 1.0193 -2.0032 3.1227 1512 0.171

regimePolygamy -0.2294 -1.9480 3.6129 2000 0.425

treatment0.5OD -0.9935 -2.2559 0.2498 2000 0.113

treatment1OD -1.2189 -2.4438 0.1292 2000 0.072 .

block2 0.6998 -1.7666 3.3902 2000 0.282

regimePolygamy:treatment0.5OD -1.5373 -3.3340 0.3553 2000 0.098 .

regimePolygamy:treatment1OD -4.1079 -6.3302 -1.8884 2000 0.001***

**Table S4f:** Bayesian mixed effects model estimating the log counts of *P. enthomophila* in mated females from polygamous and monogamous regimes 12h post-infection.

prior_load = list(R = list(V = 1, fix = 1),

G = list(G1 = list(V = 1, nu =10^-6), G2 = list(V = 1, nu =10^-6)))

MCMC_load <- MCMCglmm(logload ~ regime*treatment + block,

+ random = ~ line + treatment:line,

+ rcov = ~ units, data = data.frame(load),

+ family = *"gaussian"*, prior = prior_load, nitt=1050000,

+ slice=TRUE, burnin=50000, thin=500, verbose = FALSE,

+ pr=F)

Iterations = 50001:1049501

Thinning interval = 500

Sample size = 2000

DIC: 190.6991

G-structure: ~line

post.mean l-95% CI u-95% CI eff.samp

line 105.6 9.443e-08 0.7772 2000

~treatment:line

post.mean l-95% CI u-95% CI eff.samp

treatment:line 0.133 1.23e-07 0.1376 2000

R-structure: ~units

post.mean l-95% CI u-95% CI eff.samp

units 1 1 1 0

Location effects: logload ~ regime * treatment + block

post.mean l-95% CI u-95% CI eff.samp pMCMC

(Intercept) 3.1410 2.4566 4.2033 2000 0.012 *

regimePolygamy 0.3206 -0.7685 1.4896 2000 0.476

treatment1OD 0.2361 -0.6107 0.9527 2000 0.509

block2 0.2469 -0.8640 0.8081 2000 0.633

regimePolygamy:treatment1OD -0.6714 -1.7594 0.5098 2000 0.204

**Supplement 5: Macroevolutionary change and sexually antagonistic coevolution between male genital morphology and female PO-activity**

**Figure S5a:** Macroevolutionary change in sexual dimorphism in PO-activity across seed beetle linages.

Species codes are: robi = *Amblycerus robinae*; subf = *Zabrotes subfasciatus*; obte = *Acanthoscelides obtectus*; atro = *Bruchidius atrolineatus*; dich = *Bruchidius dichrostachydis*; tonk = *Megabruchidius tonkineus*; dors = *Megabruchidius dorsalis*; phas = *Callosobruchus phaseoli*; chin = *Callosobruchus chinensis*; subi = *Callosobruchus subinnotatus*; macu = *Callosobruchus maculatus*; anal = *Callosobruchus analis*.

**
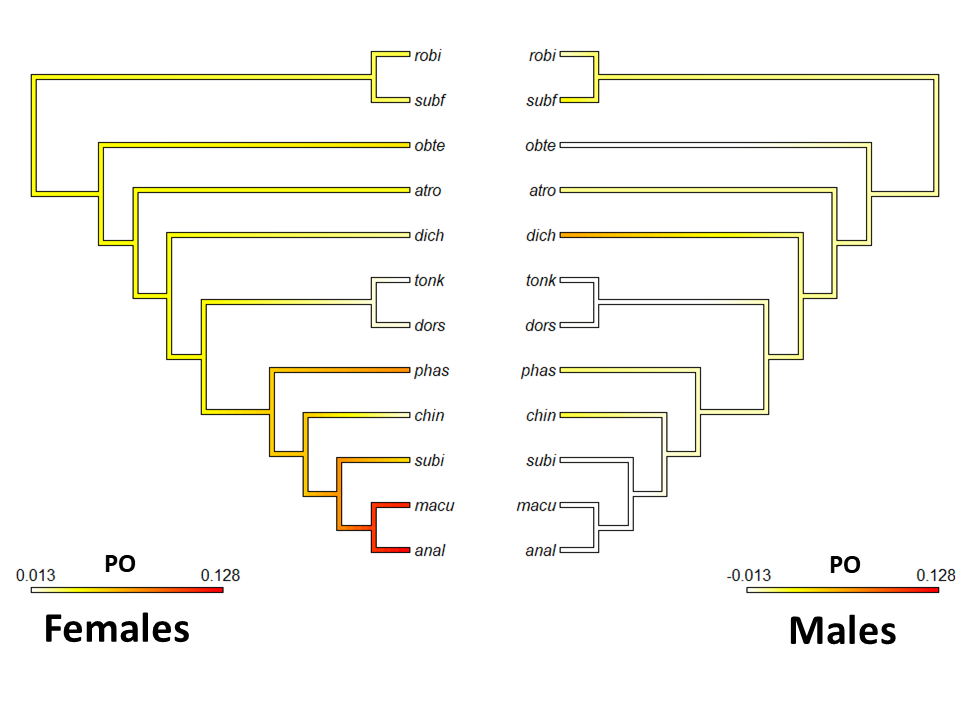
**

**Figure S5b:** Photos of genitalia rated as least and most harmful.

**
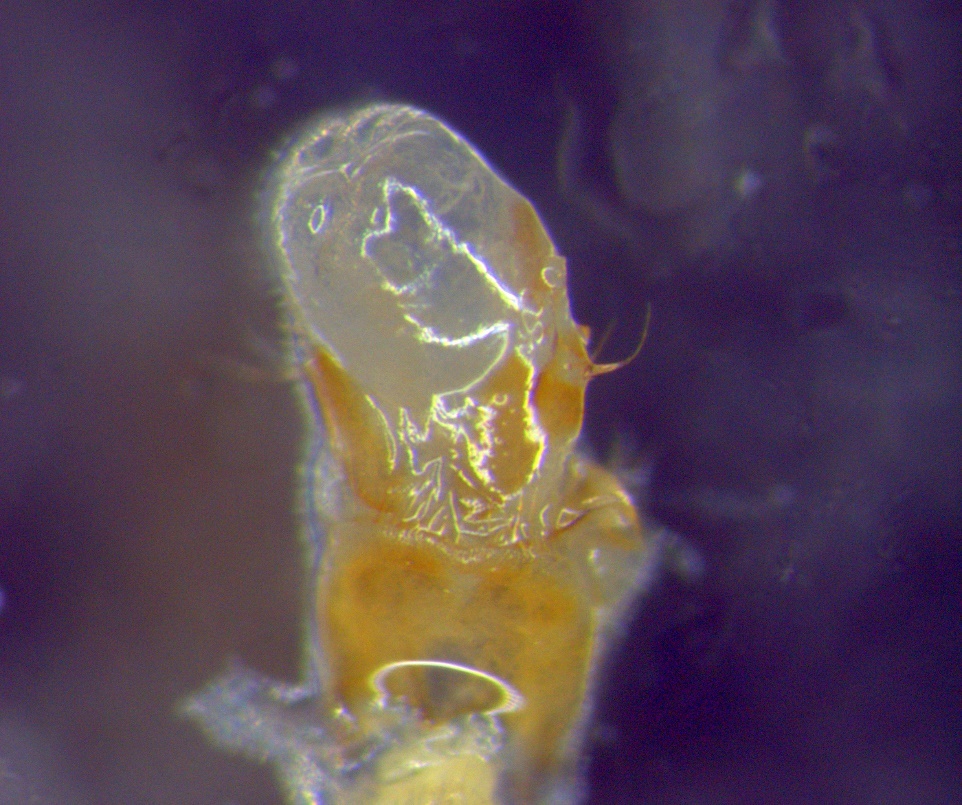

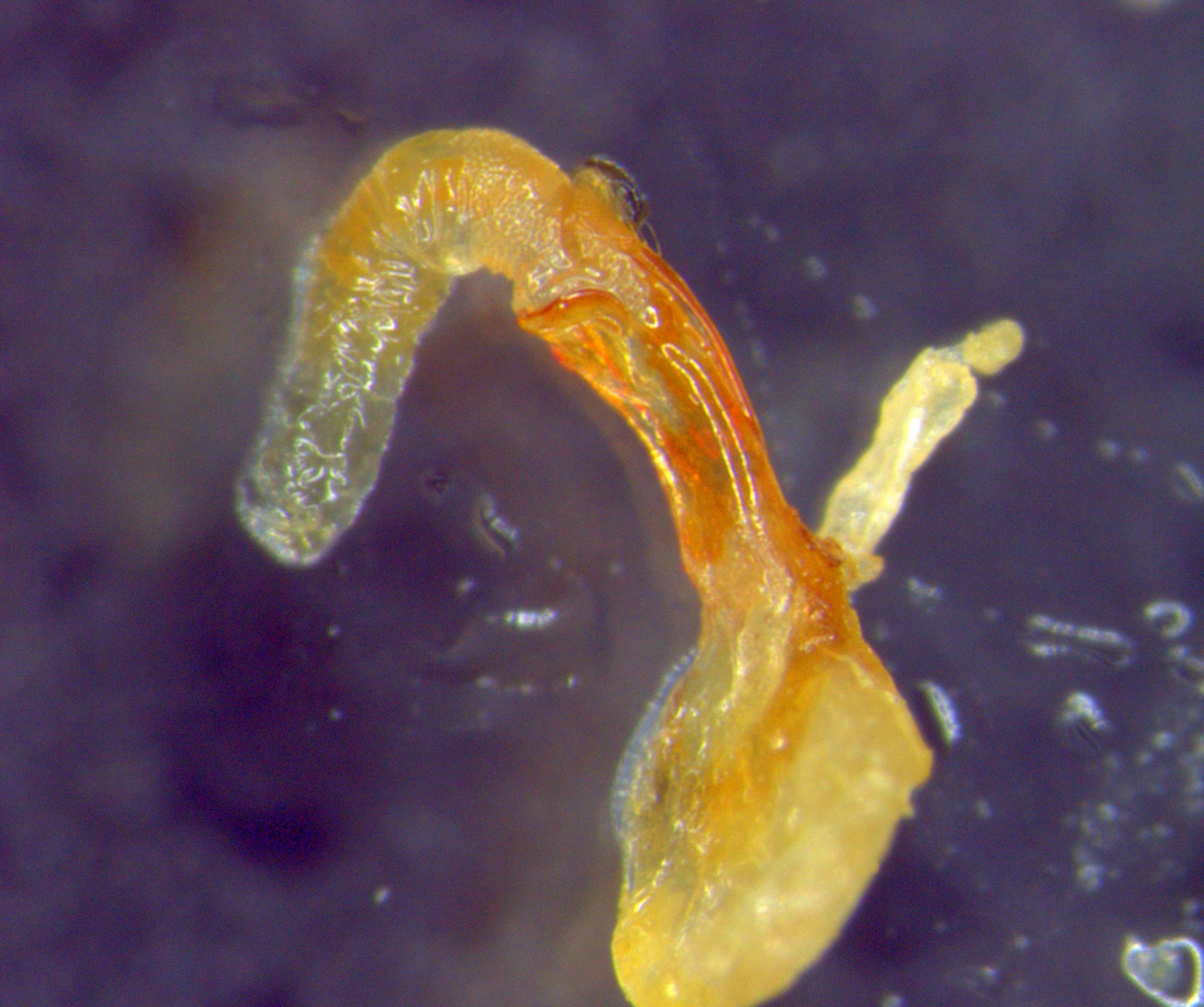
**

**Least harmful** (left: *Callosobruchus chinensis*; right: *Megabruchidius dorsalis*).

**
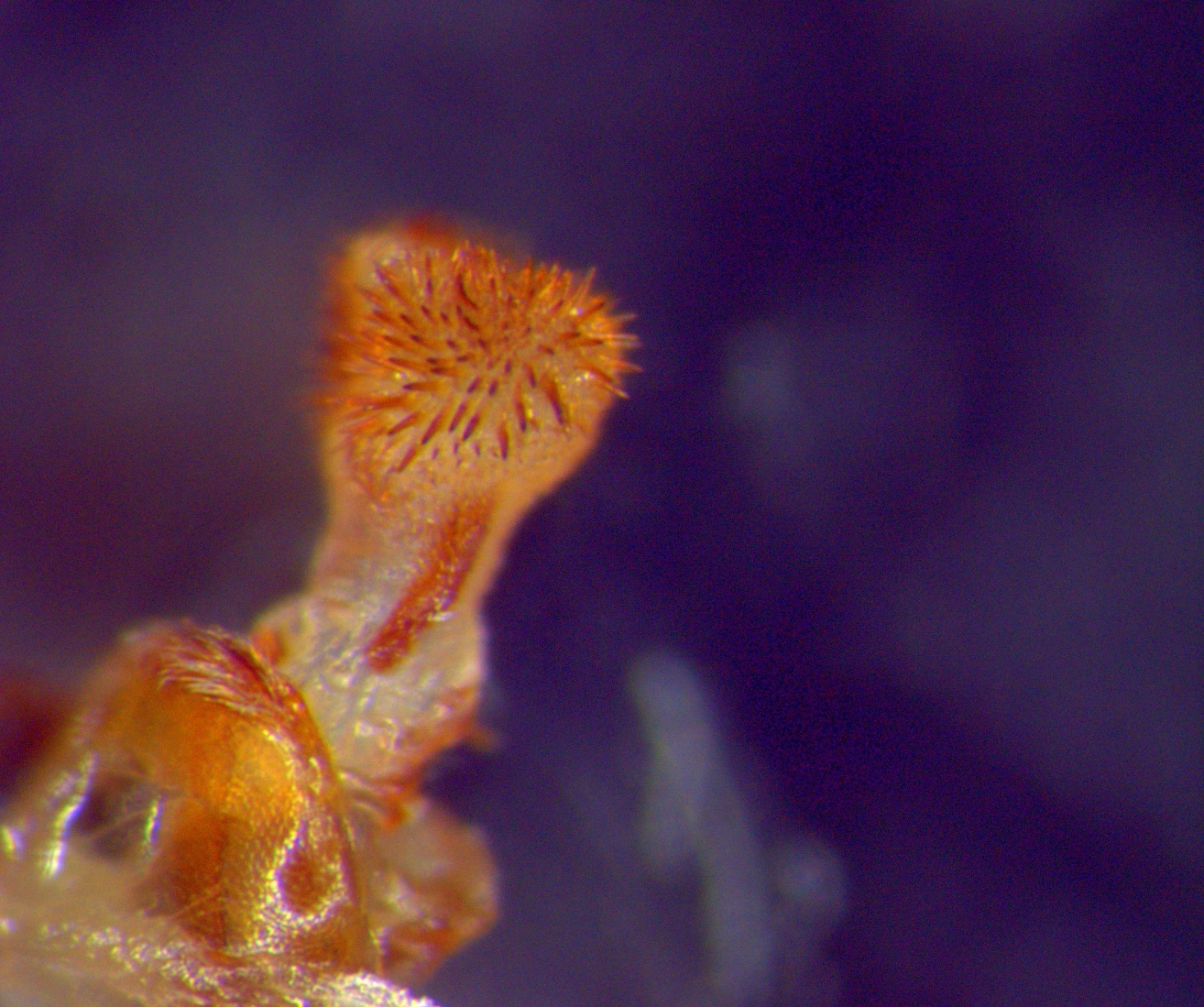

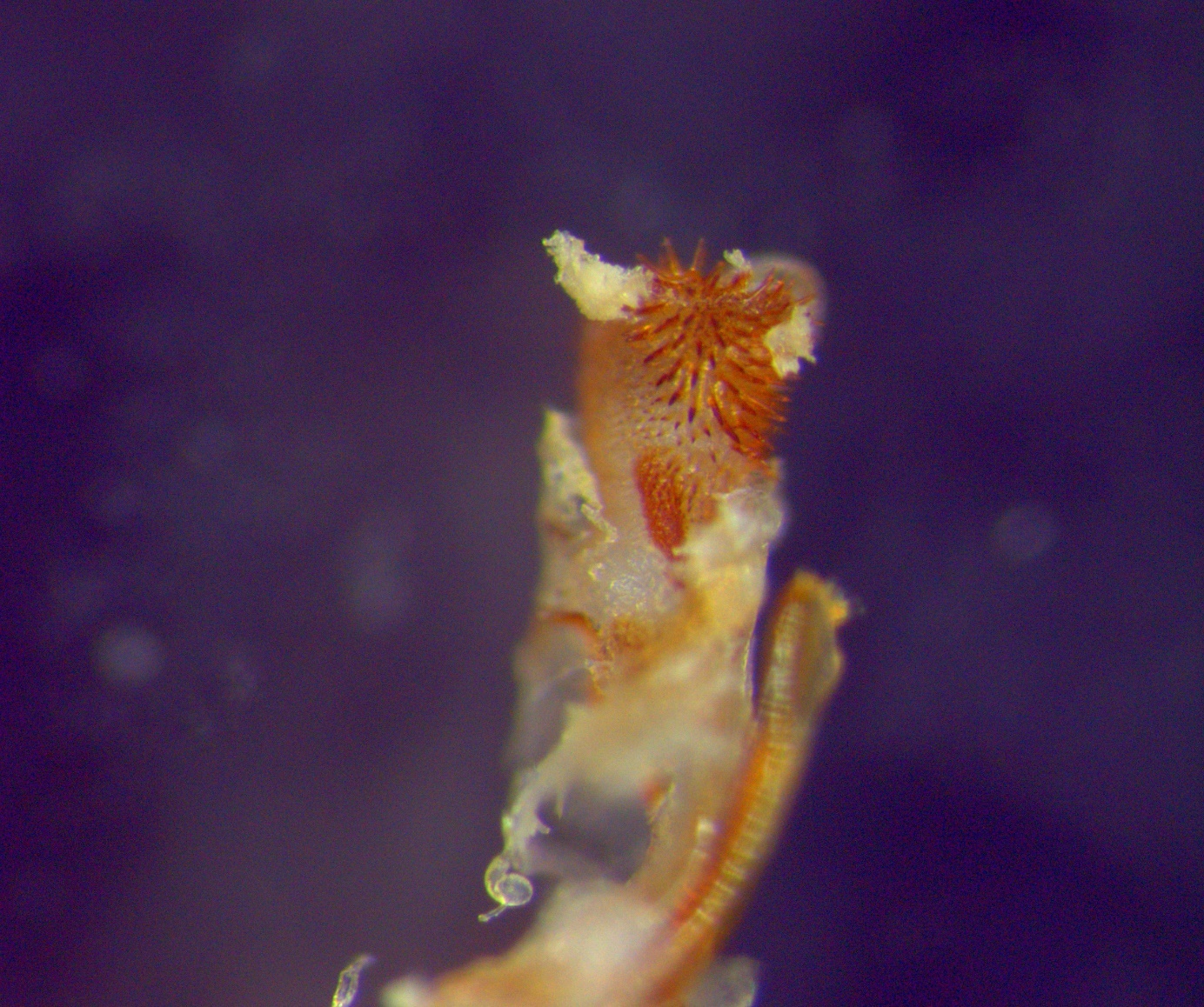
**

**Most harmful** (left: *Callosobruchus maculatus*; right: *Callosobruchus analis*).

**Table S5a:** PGLS with Ornstein-Uhlenbeck correction between male genital morphology and female PO-activity.

Generalized least squares fit by REML

Model: scale(PO.corr) ~ scale(genitalia)

AIC BIC logLik

27.04717 28.25751 -9.523586

Correlation Structure: corMartins

Formula: ~1

Parameter estimate(s):

alpha

6.703784

Coefficients:

Value Std.Error t-value p-value

(Intercept) -0.0004046 0.1586664 -0.002550 0.9980

scale(genitalia) 0.8268925 0.1643090 5.032545 0.0005

Residual standard error: 0.5061646

Degrees of freedom: 12 total; 10 residual

**Table 5b:** PGLS with Ornstein-Uhlenbeck correction between male genital morphology and male PO-activity.

Generalized least squares fit by REML

Model: scale(PO.corrM) ~ scale(genitalia)

AIC BIC logLik

37.50322 38.71356 -14.75161

Correlation Structure: corMartins

Formula: ~1

Parameter estimate(s):

alpha

2.918102

Coefficients:

Value Std.Error t-value p-value

(Intercept) 0.0753423 0.3381249 0.222824 0.8282

scale(genitalia) -0.5679619 0.2901395 -1.957548 0.0788

Residual standard error: 0.9206185

Degrees of freedom: 12 total; 10 residual
